# Supplementary material for: Climate envelope predictions indicate an enlarged suitable wintering distribution for Great Bustards (Otis tarda dybowskii) in China for the 21st century
Source: PeerJ. 2016 Feb 1;4:e1630. doi: 10.7717/peerj.1630 (PMC4741084; doi:10.7717/peerj.1630)
Supplement: Supplemental Information 2 — Supplement S2 predictors information. [file peerj-04-1630-s002.docx]

Supplement S2

| Environmental  Layers | Description | Source | Website |
| --- | --- | --- | --- |
| Bio_1 | Annual Mean Temperature ($℃$) | WorldClim | http://www.worldclim.org/ |
| Bio_2 | Mean of monthly (max temp - min temp)  ($℃$) | WorldClim | http://www.worldclim.org/ |
| Bio_3 | Isothermality (BIO2/BIO7) ( *100$℃$) | WorldClim | http://www.worldclim.org/ |
| Bio_4 | Temperature Seasonality(standard deviation *100$℃$) | WorldClim | http://www.worldclim.org/ |
| Bio_5 | Max Temperature of Warmest Month ($℃$) | WorldClim | http://www.worldclim.org/ |
| Bio_6 | Min Temperature of Coldest Month ($℃$) | WorldClim | http://www.worldclim.org/ |
| Bio_7 | Temperature Annual Range (BIO5-BIO6) ($℃$) | WorldClim | http://www.worldclim.org/ |
| Bio_8 | Mean Temperature of Wettest Quarter ($℃$) | WorldClim | http://www.worldclim.org/ |
| Bio_9 | Mean Temperature of Driest Quarter ($℃$) | WorldClim | http://www.worldclim.org/ |
| Bio_10 | Mean Temperature of Warmest Quarter ($℃$) | WorldClim | http://www.worldclim.org/ |
| Bio_11 | Mean Temperature of Coldest Quarter ($℃$) | WorldClim | http://www.worldclim.org/ |
| Bio_12 | Annual Precipitation (mm) | WorldClim | http://www.worldclim.org/ |
| Bio_13 | Precipitation of Wettest Month (mm) | WorldClim | http://www.worldclim.org/ |
| Bio_14 | Precipitation of Driest Month (mm) | WorldClim | http://www.worldclim.org/ |
| Bio_15 | Precipitation Seasonality (mm) | WorldClim | http://www.worldclim.org/ |
| Bio_16 | Precipitation of Wettest Quarter (mm) | WorldClim | http://www.worldclim.org/ |
| Bio_17 | Precipitation of Driest Quarter (mm) | WorldClim | http://www.worldclim.org/ |
| Bio_18 | Precipitation of Warmest Quarter (mm) | WorldClim | http://www.worldclim.org/ |
| Bio_19 | Precipitation of Coldest Quarter (mm) | WorldClim | http://www.worldclim.org/ |
| Altitude | Altitude (m) | WorldClim | http://www.worldclim.org/ |
| Aspect | Aspect(°) | Derived from Alitude | http://www.worldclim.org/ |
| Slope | Slope | Derived from Alitude | http://www.worldclim.org/ |
| Landcover | Land Cover | ESA | http://www.esa-landcover-cci.org/ |
| Disroad | Distance to roads (m) | Road layer from Natural Earth | http://www.naturalearthdata.com/ |
| Disrard | Distance to railways (m) | Rail road layer from Natural Earth | http://www.naturalearthdata.com/ |
| Disriver | Distance to Rivers (m) | River layer from Natural Earth | http://www.naturalearthdata.com/ |
| Dislake | Distance to lakes (m) | Lake layer from Natural Earth | http://www.naturalearthdata.com/ |
| Discoastline | Distance to coastline (m) | Coastline layer from Natural Earth | http://www.naturalearthdata.com/ |
| Dissettle | Distance to settlements (m) | Settle layer from Natural Earth | http://www.naturalearthdata.com/ |
