# Supplementary material for: Climate envelope predictions indicate an enlarged suitable wintering distribution for Great Bustards (Otis tarda dybowskii) in China for the 21st century
Source: PeerJ. 2016 Feb 1;4:e1630. doi: 10.7717/peerj.1630 (PMC4741084; doi:10.7717/peerj.1630)
Supplement: Supplemental Information 5 [file peerj-04-1630-s005.docx]

Supplement S5 Ranks of variable importance

| Rank | Variable | Score |
| --- | --- | --- |
| 1 | Slope | 100.0000 |
| 2 | Land cover | 91.3057 |
| 3 | Altitude | 86.4094 |
| 4 | Distance to settlement | 71.7420 |
| 5 | Distance to coastline | 54.9795 |
| 6 | Distance to railroad | 39.9056 |
| 7 | BIO_16 | 37.0350 |
| 8 | BIO_4 | 31.2057 |
| 9 | DISLAKE | 29.9996 |
| 10 | BIO_5 | 21.8613 |
| 11 | BIO_8 | 21.7903 |
| 12 | BIO_12 | 19.6589 |
| 13 | BIO_6 | 19.2762 |
| 14 | BIO_18 | 18.8936 |
| 15 | BIO_13 | 17.1879 |
| 16 | BIO_10 | 15.9785 |
| 17 | BIO_11 | 15.5023 |
| 18 | BIO_1 | 14.0428 |
| 19 | Distance to river | 9.1855 |
| 20 | BIO_7 | 5.3056 |
| 21 | BIO_9 | 4.8755 |
| 22 | Distance to road | 4.0511 |
| 23 | BIO_15 | 3.8332 |
| 24 | BIO_3 | 3.7084 |
| 25 | Aspect | 2.7665 |
| 26 | BIO_2 | 1.5240 |
| 27 | BIO_17 | 1.1440 |
| 28 | BIO_19 | 1.1238 |
| 29 | BIO_14 | .2668 |
