# Supplementary material for: Climate envelope predictions indicate an enlarged suitable wintering distribution for Great Bustards (Otis tarda dybowskii) in China for the 21st century
Source: PeerJ. 2016 Feb 1;4:e1630. doi: 10.7717/peerj.1630 (PMC4741084; doi:10.7717/peerj.1630)
Supplement: Supplemental Information 6 [file peerj-04-1630-s006.docx]

**Supplement S6 Partial dependence plots of each variable**

**
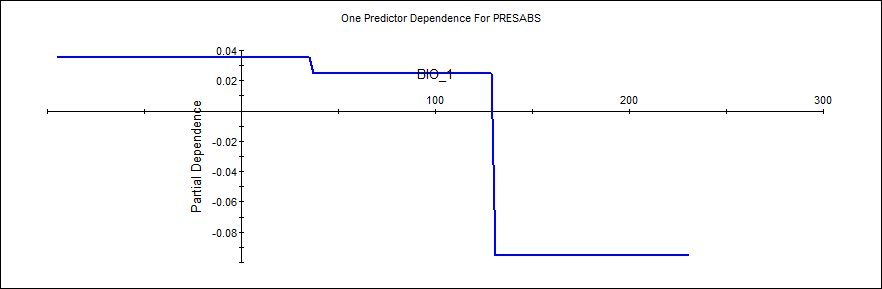
**

**Bio_1**

**
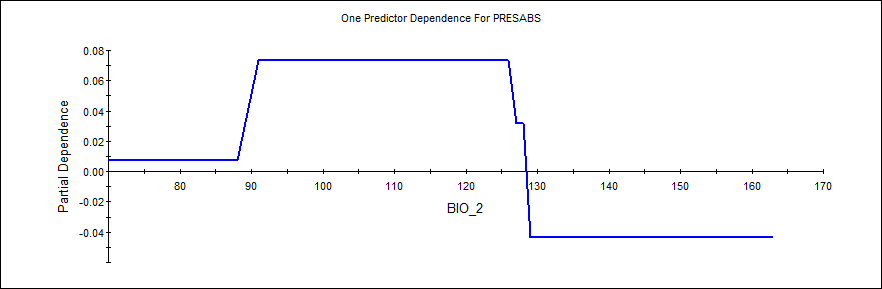
**

**Bio_2**

**
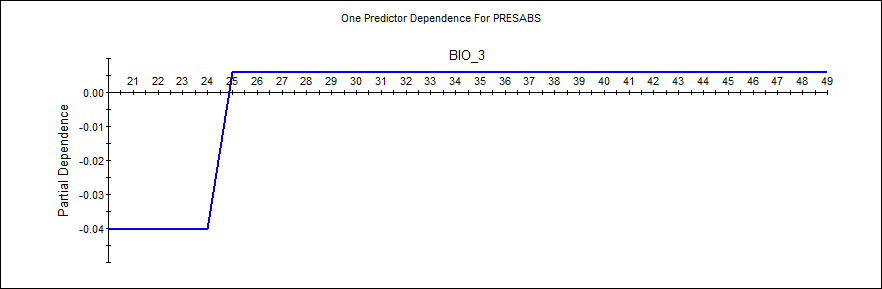
**

**Bio_3**

**
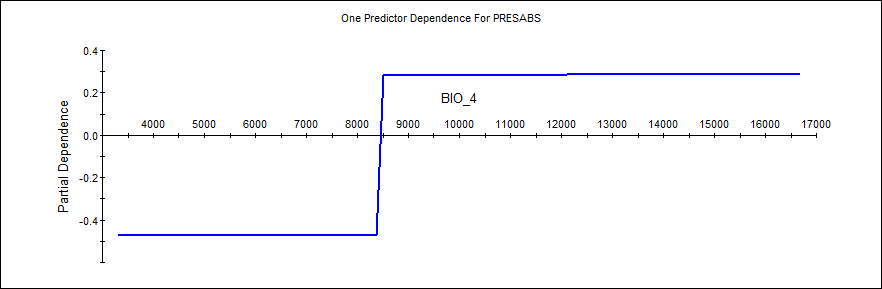
**

**Bio_4**

**
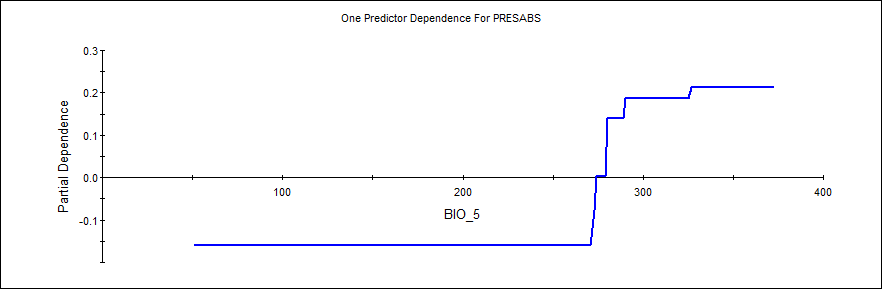
**

**Bio_5**

**
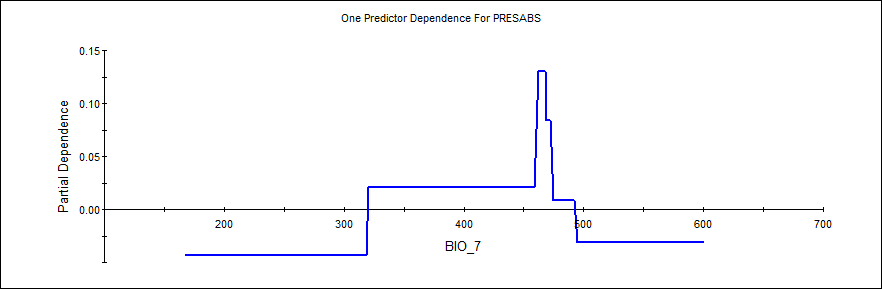
**

**Bio_7**

**
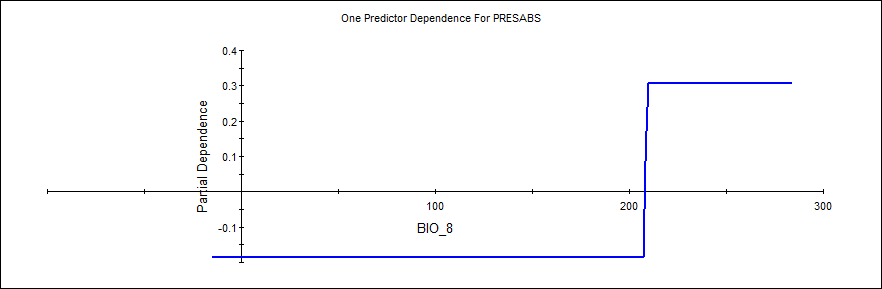
**

**Bio_8**

**
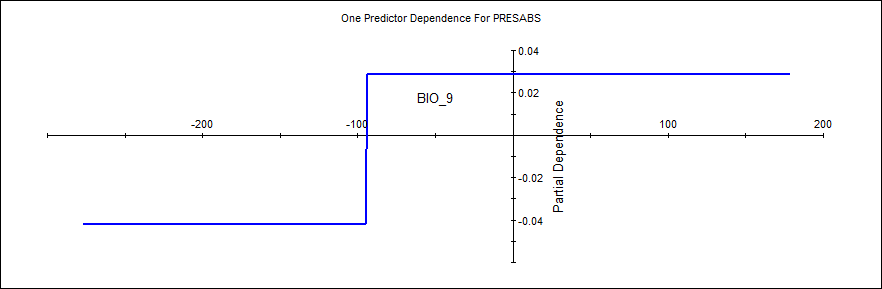
Bio_9**

**
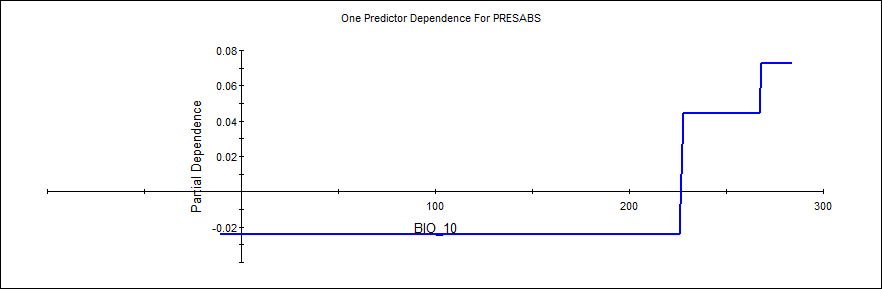
**

**Bio_10**

**
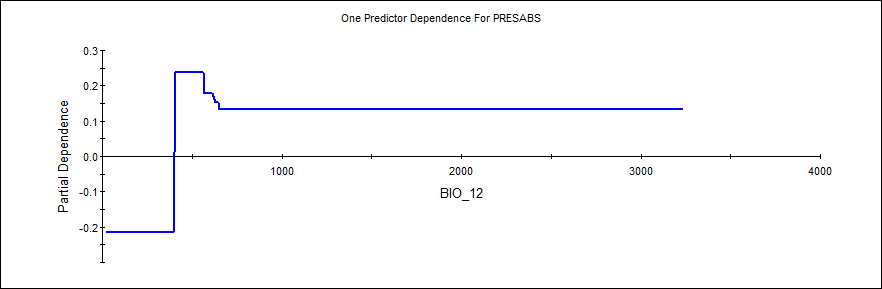
**

**Bio_12**

**
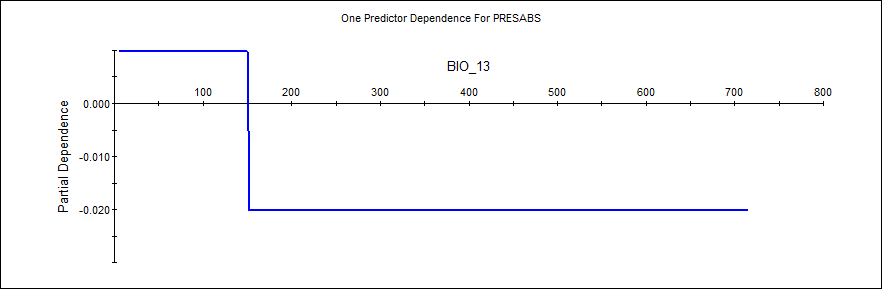
**

**Bio_13**

**
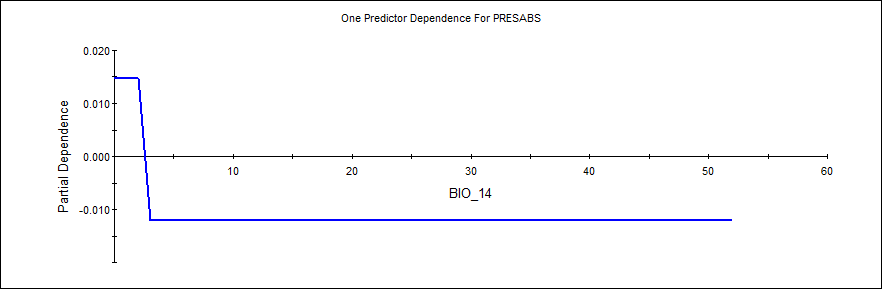
Bio_14**

**
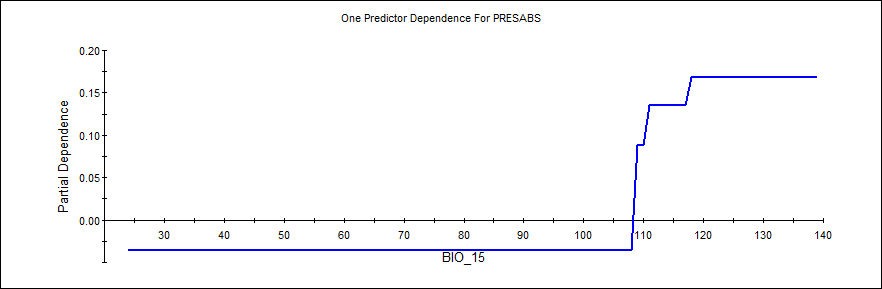
**

**Bio_15**

**
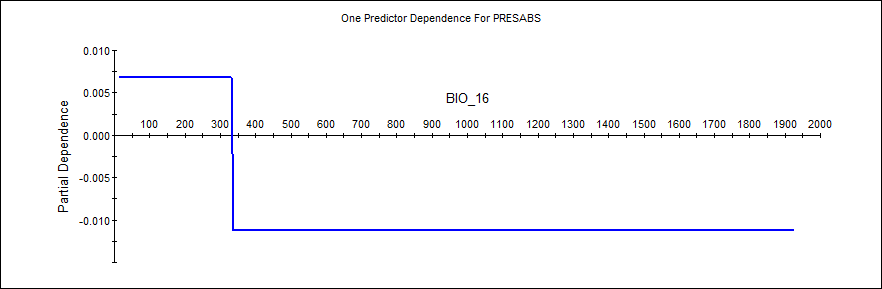
**

**Bio_16**

**
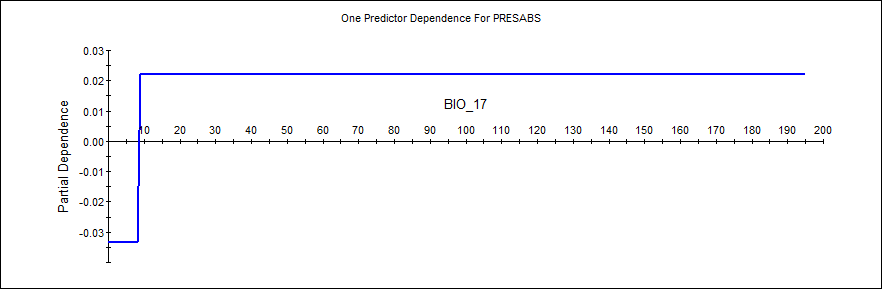
**

**Bio_17**

**
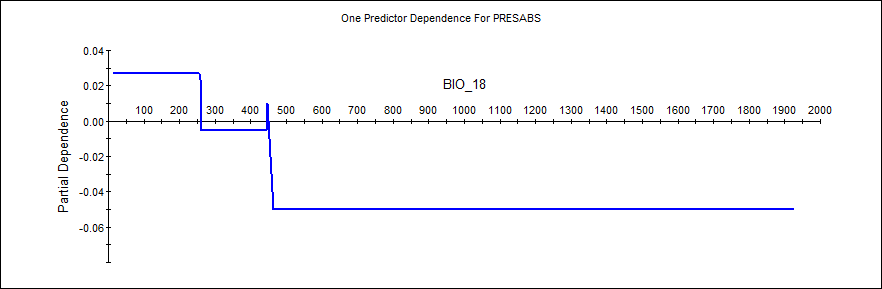
**

**Bio_18**

**
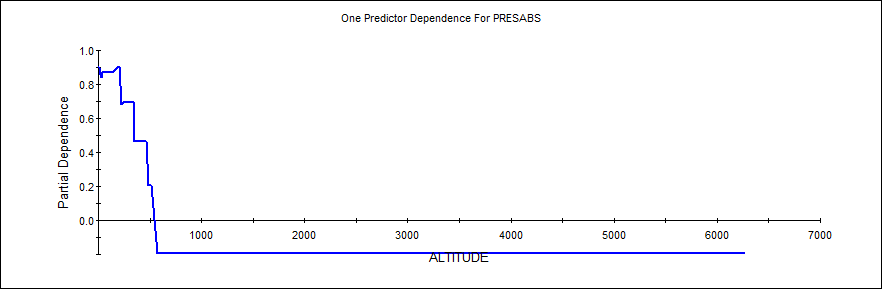
**

**Altitude**

**
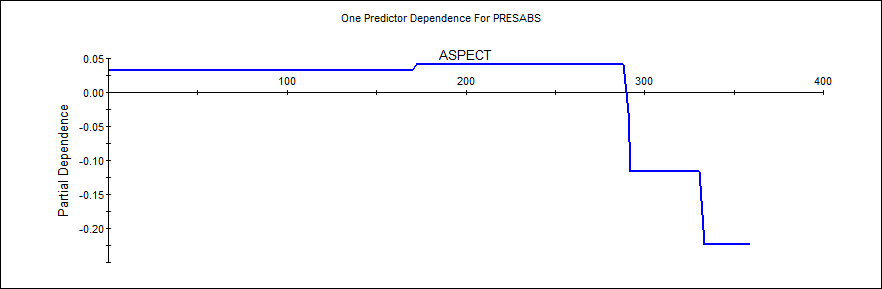
**

**Aspect**

**
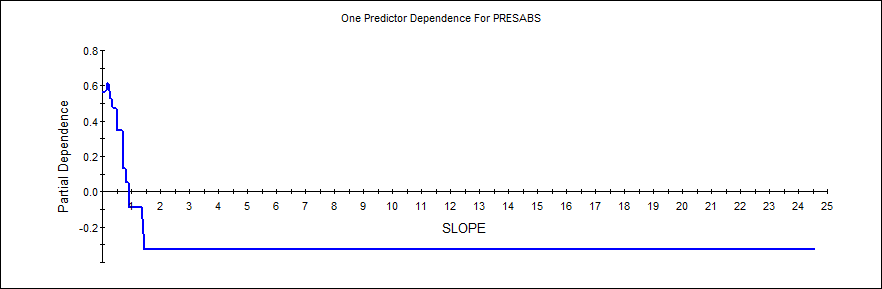
**

**Slope**

**
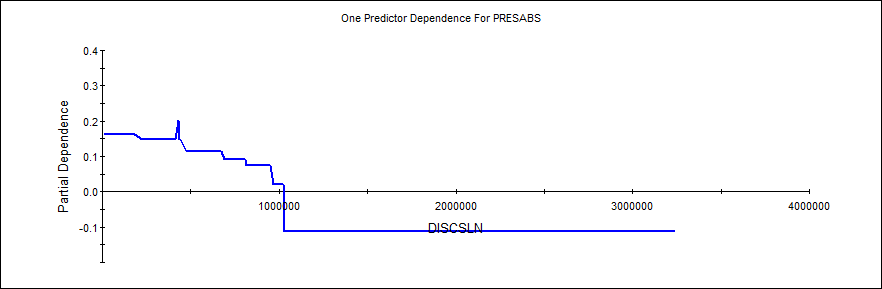
**

**Distance to coastline
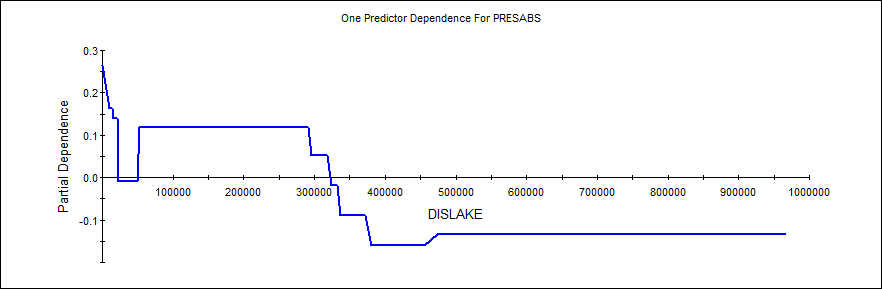
**

**Distance to lake**

**
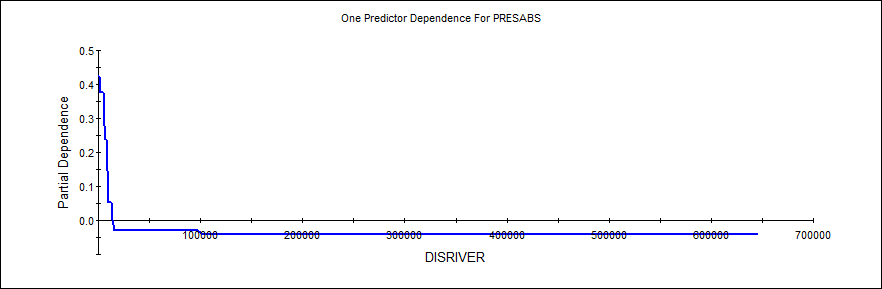
**

**Distance to river**

**
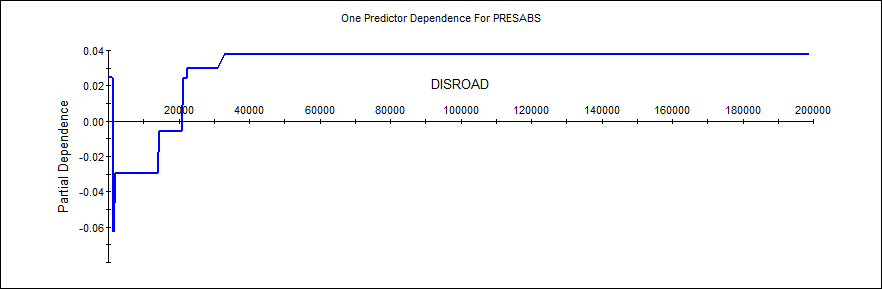
**

**Distance to road**

**
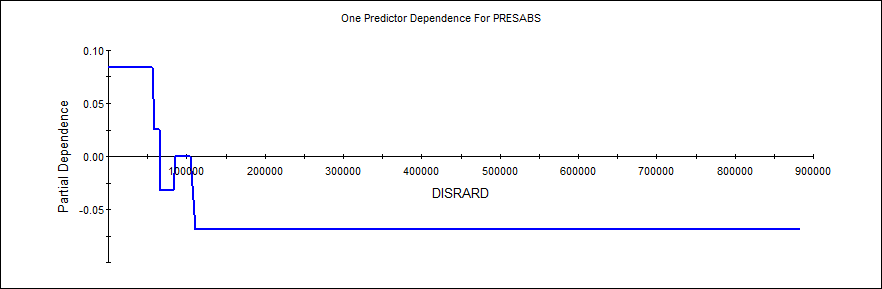
**

**Distance to rail road**

**
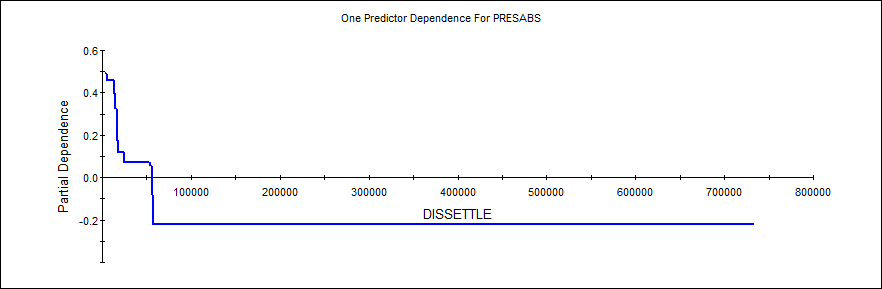
**

**Distance to settlement**

**
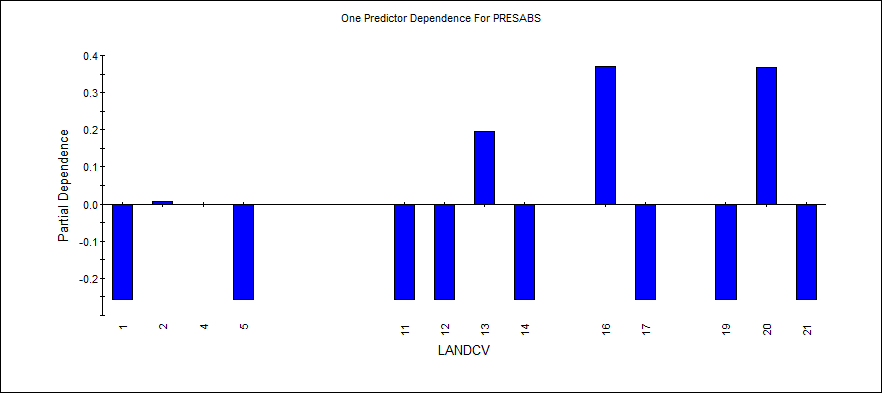
**

**Land cover**

**The legend of land cover layer**

|  | GLC Global Class (according to LCCS terminology) |
| --- | --- |
|  | Tree Cover, broadleaved, evergreen  *LCCS >15% tree cover, tree height >3m*  (Examples of sub-classes at regional level* :  *closed > 40% tree cove; open 15-40% tree cover)* |
|  |  |
|  | Tree Cover, broadleaved, deciduous, closed |
|  | Tree Cover, broadleaved, deciduous, open  *(open 15-40% tree cover)* |
|  | Tree Cover, needle-leaved, evergreen |
|  | Tree Cover, needle-leaved, deciduous |
|  | Tree Cover, mixed leaf type |
|  | Tree Cover, regularly flooded, fresh water (& brackish) |
|  | Tree Cover, regularly flooded, saline water,  (daily variation of water level) |
|  | Mosaic:  Tree cover / Other natural vegetation |
|  | Tree Cover, burnt |
|  | Shrub Cover, closed-open, evergreen  (Examples of sub-classes at reg. level *: (i) sparse tree layer) |
|  | Shrub Cover, closed-open, deciduous  (Examples of sub-classes at reg. level *: (i) sparse tree layer) |
|  | Herbaceous Cover, closed-open  (Examples of sub-classes at regional level *:  (i) natural, (ii) pasture, (iii) sparse trees or shrubs) |
|  | Sparse Herbaceous or sparse Shrub Cover |
|  | Regularly flooded Shrub and/or Herbaceous Cover |
|  | Cultivated and managed areas  (Examples of sub-classes at reg. level *:  (i) terrestrial; (ii) aquatic (=flooded during cultivation), and under terrestrial: (iii) tree crop & shrubs (perennial), (iv) herbaceous crops (annual), non-irrigated, (v) herbaceous crops (annual), irrigated) |
|  |  |
|  |  |
|  |  |
|  |  |
|  | Mosaic:  Cropland / Tree Cover / Other natural vegetation |
|  | Mosaic:  Cropland / Shrub or Grass Cover |
|  | Bare Areas |
|  | Water Bodies (natural & artificial) |
|  | Snow and Ice (natural & artificial) |
|  | Artificial surfaces and associated areas |
